# Non-Linear Association between Exposure to Ambient Temperature and Children’s Hand-Foot-and-Mouth Disease in Beijing, China

**DOI:** 10.1371/journal.pone.0126171

**Published:** 2015-05-26

**Authors:** Meimei Xu, Weiwei Yu, Shilu Tong, Lei Jia, Fengchao Liang, Xiaochuan Pan

**Affiliations:** 1 Department of Occupational and Environmental Health, School of Public Health, Peking University, Beijing 100191, China; 2 Epidemiology and Biostatistics Discipline, School of Population Health, University of Queensland, Brisbane, QLD 4006, Australia; 3 School of Public Health and Social Work, Queensland University of Technology, Kelvin Grove, Brisbane, QLD 4059, Australia; 4 Institute for Infectious Disease and Endemic Disease Control, Beijing Center for Disease Prevention and Control, Beijing 100013, China; The Ohio State University, UNITED STATES

## Abstract

**Background:**

Hand, foot and mouth disease (HFMD) was listed as a notifiable communicable disease in 2008 and is an emerging public health problem in China, especially for children. However, few data are available on the risk assessment of the potential reasons for HFMD in Beijing. This study examined the association of temperature with the incidence of children’s HFMD in Beijing at the daily scale for the first time.

**Methods:**

A newly developed case-crossover design with a distributed lag nonlinear model (DLNM) was used to assess the delayed and cumulative associations of daily temperature with gender- and age-specific HFMD in Beijing, China, during 2010–2012. Relative humidity, day of the week, public holiday, season and long-term trends were controlled in the model.

**Results:**

Among the total of 113,475 cases, the ratio between males and females was 1.52:1. HFMD was more prevalent in May-July. The temperature-HFMD relationships were non-linear in most age groups except for children aged 6–15 years, with a peak at 25.0~27.5°C. The high-temperature risks were greater, appeared earlier and lasted longer than the low-temperature risks. The relative risks for female children and those aged 6–15 years were higher than those among other groups.

**Conclusion:**

Rising temperatures increased the incidence of children’s HFMD, with the largest association at 25.0~27.5°C. Females and children aged 6–15 years were more vulnerable to changes in temperature with regard to the transmission of HFMD than males and other age groups, respectively. Further studies are warranted to confirm these findings in other populations.

## Introduction

Hand, foot and mouth disease (HFMD) is a common viral illness mainly caused by coxsackievirus A16 (Cox A16) and enterovirus 71(EV 71). Children under 5 years old are more susceptible to this disease, because over 50% of these children lack neutralizing antibodies against EV 71 and Cox A16[1, 2]. Symptoms of HFMD include fever, headache and poor appetite, followed by intense sore throat and a rash with very small blisters on hands, feet, and diaper area. Few may develop severe symptoms, such as encephalitis, meningitis and myocarditis. HFMD may be spread through close personal contact, the air, and contact with faecal matter, including contaminated objects and surfaces. So far there is no vaccine or specific treatment for HFMD.

Since its first incursion in 1957, HFMD has been reported worldwide, especially in the Western Pacific Region [3–6]. Since 2008, HFMD has been classified as a notifiable infectious disease according to the Law of the People’s Republic of China on Prevention and Treatment of Infectious Diseases. In recent years, the prevalence of HFMD has been increasing and become one of very common infectious diseases in some areas of China. There were 1,774,669 cases of HFMD and 905 deaths in whole China in 2010, about four times higher than that (488,955 cases and 126 deaths) in 2008 [7]. The increasing phenomenon has made Chinese government concern about the risk factors of HFMD prevalence and seek related control approaches.

Previous studies [3, 8, 9] have observed the seasonality of HFMD, e.g. epidemic peek observed in summer or late autumn, which indicated atmospheric temperature may impact the incidence of HFMD. Several epidemiological studies have tried to examine the association between temperature and HFMD [10–13], but these findings are inconsistent. Onozuka et al (2011) [12] observed that rising weekly temperature was linked to elevated HFMD incidence in Japan. Huang et al (2013)[13] found that percentage increases of weekly HFMD cases were significantly associated with the increases of weekly temperature below 25°C and insignificantly with the increases of weekly temperature above 25°C in Guangzhou, China. A study in Taiwan [14] found that the incidence of EV71 infections began to rise at temperatures above 13°C while the incidence began to decline at temperatures higher than approximately 26°C. Therefore, it is not clear whether there is a real association between temperature and HFMD, and if so, the association is linear or non-linear.

Previous studies observed the association of delayed exposure to temperature on HFMD, however they have used single-day (week) models to measure the moving average lag effects [11, 15], which may overestimate or underestimate the relative risks (RRs) of current-day (week) exposure as a result of overlapping with risks from the previous days’ exposure [16]. Consequently, the total effect of exposure to the temperature on the health outcomes cannot be adequately captured [17]. The distributed lag nonlinear model (DLNM) has been developed to estimate the non-linear effects and quantify the effects of individual lags. The overall effect can be computed by summing the log relative risks of each lag in this model. Hence, DLNM is more appropriate to estimate the total RRs of delayed exposure to temperature on HFMD.

The case-crossover design developed by Maclure[18] is useful to study the effects of short-term exposure to temperature. The case-crossover approach can adjust well for the confounding from seasonal and secular trends by matching case and control days in relatively small time windows (e.g., calendar month), however, it has a limitation on fitting nonlinear effects. The case-crossover design combined with DLNM model could more accurately estimate sophisticated nonlinear and delayed effects of temperature while controlling for season and secular trends [19], and was used in our study to examine the association of daily temperature with the incidence of children’s HFMD in temperate city of Beijing.

## Methods

### Data collection

Beijing was chosen to be the subject city of the study. It is the capital city and located in the northern tip of the roughly triangular North China Plain, where has an area of 16,410 square kilometres and a population of 20,693,000 as of 2012[20]. It has a dry, monsoon-influenced humid continental climate, characterized by hot, humid summers, and cold, windy and dry winters. Average annual temperature and precipitation was 14.0°C and 483.9 mm, respectively.

Daily data on HFMD cases in 2010–2012 were obtained from Beijing Centre for Disease Control and Prevention. HFMD cases were diagnosed based on the following symptoms: fever, vesicular lesions on hands, feet, mouth and occasionally the buttocks, which was in accordance with the National Guideline published by Chinese Ministry of Health in 2009. The HFMD cases aged 0–15 years accounting for 98.87% of all the HFMD cases were chosen in this study.

Daily data on mean temperature, relative humidity, duration of sunshine, rainfall, and wind velocity of Beijing were collected from China Meteorological Data Sharing Service System (http://cdc.cma.gov.cn). The weather monitoring station is located in Daxing District (N39° 48', E116°28') in southeast Beijing. The data from this monitoring station are always used to represent the meteorological conditions in the whole Beijing.

### Data analysis

The time-stratified case-crossover design combined with DLNM was applied to explore the impact of temperature on HFMD prevalence. A quasi-Poisson function that allows for over-dispersion in daily HFMD cases was used in the model.

$$\begin{array}{l} \;{\text{Y}}_{\text{t}}\sim \text{poisson}\;\left(\mu_{\text{t}}\right)\\ \text{Log}\left(\mu_{\text{t}}\right)=\alpha +\text{ns}\left(\;{\text{T}}_{\text{t},\text{l}},5,4\right)+\text{ns}\left(\;{\text{RH}}_{\text{t},\text{l}},5,4\right)+\\ \;\text{ns}\;\left(\text{sunshine},3\right)+\text{ns}\;\left(\text{rainfall},3\right)+\text{ns}\left(\text{wind velocity},3\right)\\ \;+\lambda \;{\text{Strata}}_{\text{t}}+\gamma \;{\text{DOW}}_{\text{t}}+\kappa \;{\text{PH}}_{\text{t}} \end{array}$$

Where t is the day of observation; Y_t_ is the observed daily HFMD case counts on day t; α is the intercept; l is the lag days. T_t,l_ and RH_t,l_ is a matrix obtained by applying cross-basis function in the DLNM to temperature and relative humidity, respectively, where ns() is a natural cubic spine. A natural cubic spline DLNM was used to model the nonlinear temperature/humidity association with 5 degrees of freedom (*df*) and the lagged association using 4 df [19]. Three df was used to smooth sunshine, rainfall and wind velocity [12,13]. Strata_t_ is a categorical variable of the year and calendar month used to control for long-term trend and seasonality, and λ is a vector of coefficients. DOW_t_ is day of the week on day t, and γ is a vector of coefficients. PH_t_ is a binary variable that is “1” if day t was a public holiday, and κ is the coefficient.

Rainfall and wind velocity were insignificantly associated with HFMD in our model, thus the two variables were removed from the model.

A maximum lag of 13 days was used to explore the potential lag associations of temperature and relative humidity in our model for eliminating the role of the natural incubation period (approximately 3 to 7 days) [21]. Spline knots were placed at equal spaces in the temperature/humidity range, and at equal intervals in the log scale of lags using the default setting of DLNM. The median value of temperature/humidity was defined as the reference value for calculating relative risks.

The initial analysis observed that the exposure-response curve was non-linear, with an inverted V-shape. We also found the RRs of low temperature were smaller than RRs of high temperature. Thus, we redefined the lowest temperature of -12.5°C in this study as the reference value for calculating relative risks. With the purpose of evaluating the characteristics of the temperature-HFMD relationship, the relative risks of HFMD were estimated by different temperature structures (1^st^, 25^th^, 50^th^, 75^th^, 99^th^ percentile and the cut-off point) relative to the reference value.

Stratified analyses were conducted for both male and female children, and different age groups (<1, 1–3, 3–6, and 6–15 years). The age groups were classified based on the different daily activity and environment. In China, children aged 0–3 years are all cared at home, but the group aged <1 years are different from that aged 1–3 years in daily activities. The group aged 3–6 years usually attend kindergartens and 6–15 years go to school.

Sensitive analyses were performed by changing the maximum lags (13 days) to 20 days for the DLNM and the df for weather variables from 4 to 7.

This study did not involve informed consent as patient records/information was anonymized and de-identified before we obtained the data.

All statistical analyses were performed in statistical software R version 3.0.1 (R Development Core Team, 2013). The “dlnm” package was used to create the DLNM model. The statistical tests for Spearman correlations were two-sided and P-values with less than 0.05 were considered statistically significant. All risk estimates were presented with corresponding 95% confidence intervals.

## Results

Table 1 shows the summary statistics for age- and gender-specific incidences of the children’s HFMD and the weather variables during the study period. There were totally 113,475 HFMD cases aged 0–15 years in Beijing between 1 January 2010 and 31 December 2012, of which 6.14% were aged <1 year, 41.91% aged 1–3 years, 44.84% aged 3–6 years, and 7.11% aged 6–15 years. The ratio between male and female cases was 1.52:1. The daily average for the temperature, relative humidity, sunshine, wind velocity and rainfall were 13.0°C (range: -12.5~34.5°C), 50.4% (range: 9.0%~97.0%), 6.7h (range: 0~13.8h), 2.2m/s (range: 0.6~6.4m/s) and 1.8mm (range: 0.0~82.9 mm), respectively.

**Table 1:** Descriptive statistics for daily data on meteorological variables and children’s HFMD cases in Beijing, 2010–2012.

	n	Mean	SD	Min.	Median	Max.
Cases						
Daily HFMD cases	1084*	104.7	111.4	0	64	483
Daily HFMD cases in male	1084*	63.1	67.2	0	39	297
Daily HFMD cases in female	1084*	41.6	44.6	0	25	192
Daily HFMD cases aged <1 years	1084*	6.4	8	0	3	44
Daily HFMD cases aged 1–3 years	1084*	43.9	46.3	0	26	202
Daily HFMD cases aged 3–6 years	1084*	46.9	51.2	0	29	265
Daily HFMD cases aged 6–15 years	1084*	7.4	9.6	0	4	53
Meteorological variable						
Temperature (°C)	1096	13	11.7	-12.5	14.8	34.5
Relative humidity (%)	1096	50.4	20.3	9	52	97
Sunshine (hour)	1096	6.7	4.1	0	7.8	13.8
Wind velocity(m/s)	1096	2.2	0.9	0.6	2.1	6.4
Rainfall(mm)	1096	1.8	7.8	0	0	82.9

n: the number of days

* means that HFMD data for 12 days were missing.

**Fig 1:**
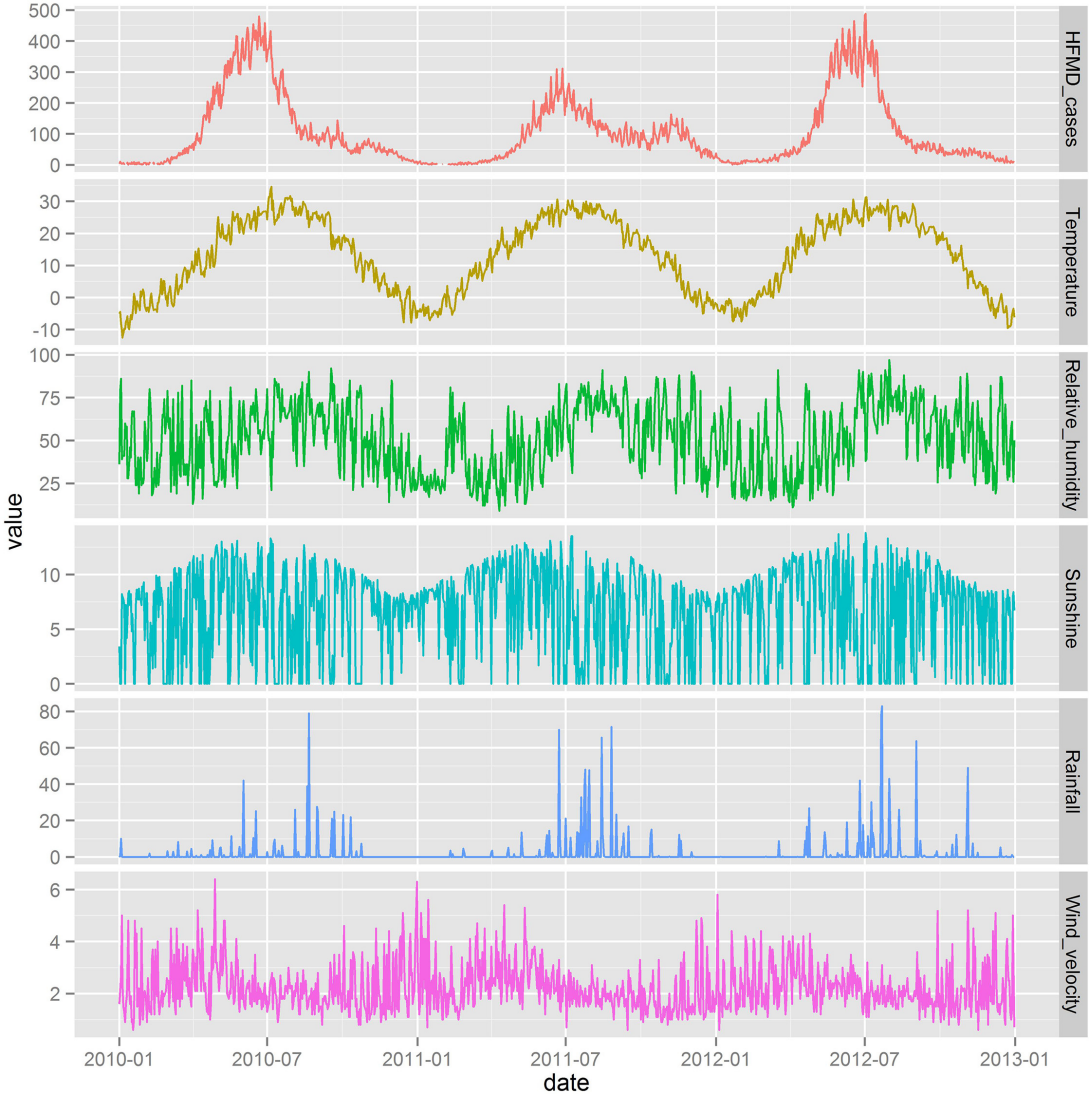
Daily distribution of meteorological variables and children’s HFMD cases in Beijing, 2010–2012.

Fig 1 illustrates the daily distribution of HFMD cases and meteorological variables during the study period, indicating a seasonal pattern. The number of HFMD cases was the lowest in year 2011. A peak was observed in May-July, along with the second small peak around November of 2011 (Fig 1).

Table 2 reveals the Spearman correlations between daily meteorological variables and HFMD cases. We found that the HFMD incidence was positively associated (p<0.01) with temperature (r = 0.83), relative humidity (r = 0.37), sunshine (r = 0.09) as well as rainfall (r = 0.22), but not significantly with wind velocity (P>0.05). Rainfall and wind velocity had strong correlation with relative humidity and sunshine, validating the lack of association between rainfall/wind velocity and HFMD in our model.

**Table 2:** Spearman correlation between daily meteorological variables and children’s HFMD cases in Beijing, 2010–2012.

	Temperature	Relative humidity	Sunshine	Rainfall	Wind velocity
Relative humidity	0.39*				
Sunshine	0.16*	-0.57*			
Rainfall	0.18*	0.47*	-0.34*		
Wind velocity	-0.01	-0.45*	0.27*	0.03	
HFMD cases	0.83*	0.37*	0.09*	0.22*	-0.01

* P<0.01

**Fig 2:**
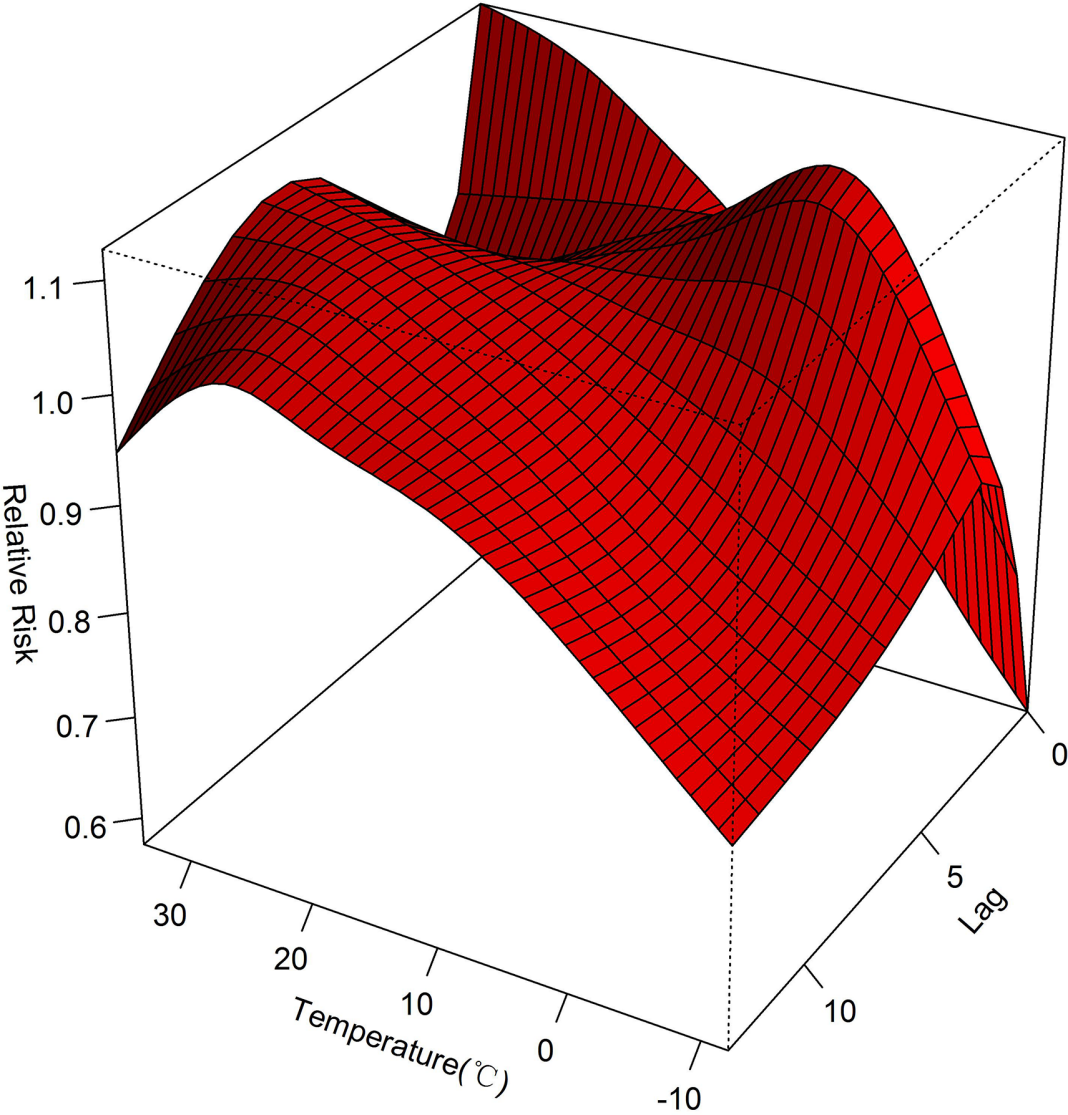
Three-dimensional plot of the relationship between mean temperature and HFMD over 13 lag days.

The three-dimensional plot shows the relationship between daily average temperature and HFMD along 13 lag days (Fig 2), with higher relative risk at the high temperature. We found that the association of temperature with HFMD may have a different lag pattern. For example, the extreme high temperatures (30.9°C) had maximum RR for HFMD cases on the current day, which subsequently decreased for the following 4 days and then turned to increase till lag 11 days; however, the extreme low temperature (-8.4°C) had the minimal RR on the current day and had the maximum RR at lag 2 days. It suggested that the associations of high temperatures appeared earlier and lasted longer time than the associations of low temperatures with HFMD incidences.

**Fig 3:**
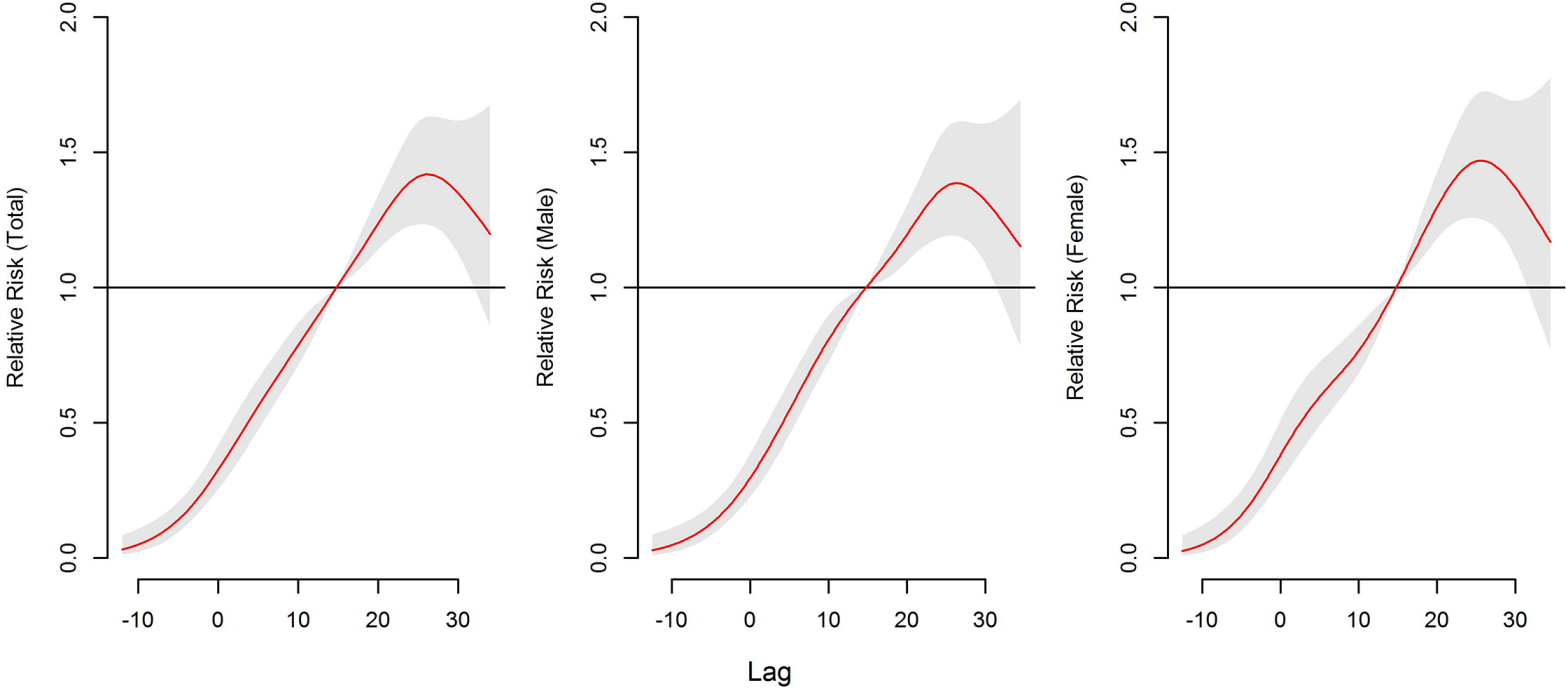
The overall relative risks of mean temperature (°C) for total and gender-specific HFMD cases over 13 days.

Fig 3 shows the association of cumulative exposure to mean temperature over 13 days with HFMD. The RRs increased with the increment of temperature and it reached the peak at 26.2°C and then began to decrease. The gender-specific RRs followed the same trends as the total RRs, with the peak at 26.4°C for male and 25.9°C for female.

**Fig 4:**
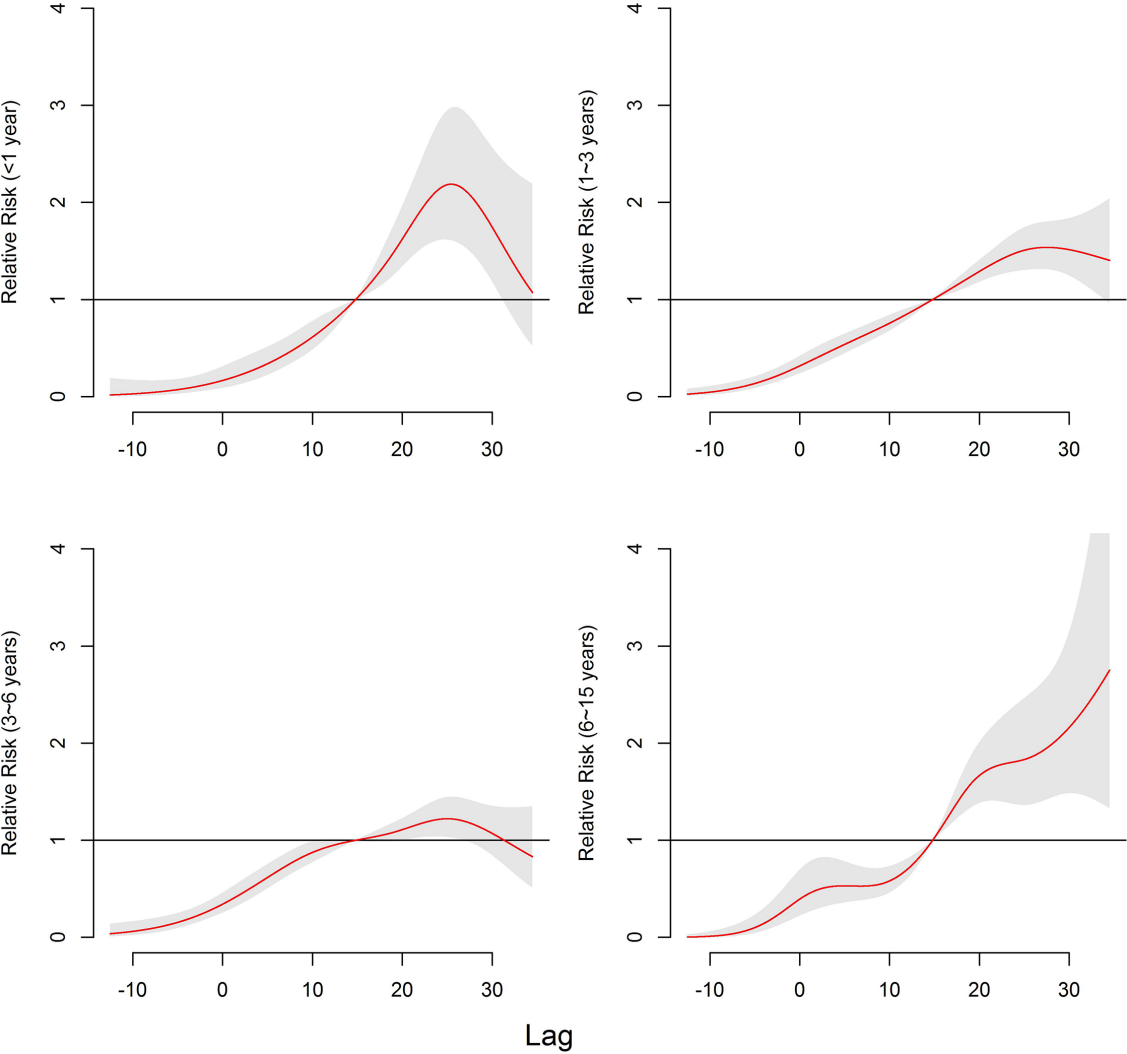
The overall relative risks of mean temperature (°C) for age-specific HFMD cases over 13 days.

Fig 4 presents the RRs of cumulative exposure to mean temperature for age-specific HFMD cases over 13 days. For children aged < 6 years, the RRs peaked at 25.4°C for those aged <1 year, 27.5°C for aged 1–3 years and 25.0°C for aged 3–6 years. For children aged 6–15 years, the exposure-response curve was approximately linear.

**Fig 5:**
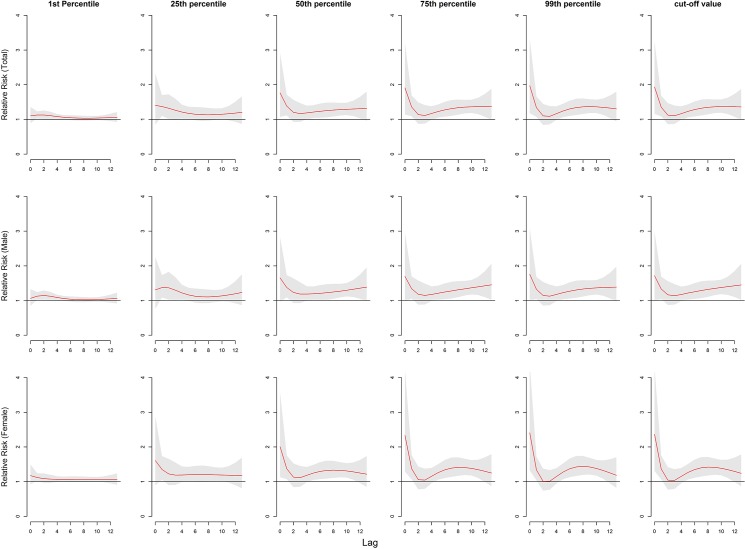
The relative risks of mean temperature (°C) for total and gender-specific HFMD cases among different lags. (Note:1st percentile:-8.4°C; 25th percentile:1.8°C; 50th percentile:14.8°C;75th percentile:24.0°C; 99th percentile:30.9°C; cut-off value for the whole subjects:26.2°C; cut-off value for male: 26.4°C; cut-off value for female: 25.9°C.)

Fig 5 summarizes the risks of different mean temperatures relative to the lowest temperature (-12.5°C) for total and gender-specific HFMD cases along the lags. For all the children, the low temperatures have smaller RRs than the high temperatures except at lag 2 day. The highest RRs occurred at the different temperature at different lag days. For example, the median of temperature had the highest RR at lag 0–6 days while 26.2°C has the highest RR at lag 0–13 days (Table 3).

**Table 3:** The relative risks of cumulative exposure to different temperatures for age-specific and gender-specific HFMD cases in Beijing.

	Temperature structure ^a^	lag0	lag0-6	lag0-13
Total	-8.4°C	1.10 (0.90, 1.35)	**1.85 (1.31, 2.62)**	**2.49 (1.61, 3.83)**
	1.8°C	1.41 (0.85, 2.32)	**5.25 (2.22, 12.42)**	**14.66 (5.08, 42.31)**
	14.8°C	**1.76 (1.07, 2.90)**	**6.09 (2.58, 14.40)**	**35.82 (12.75, 100.67)**
	24.0°C	**1.91 (1.14, 3.18)**	**6.03 (2.50, 14.51)**	**49.84 (17.45, 142.39)**
	26.2°C^b^	**1.93 (1.16, 3.23)**	**5.98 (2.49, 14.38)**	**50.83 (17.80, 145.18)**
	30.9°C	**1.97 (1.18, 3.30)**	**5.97 (2.46, 14.45)**	**47.25 (16.12, 138.50)**
Male	-8.4°C	1.06 (0.85, 1.33)	**1.84 (1.27, 2.67)**	**2.33 (1.46, 3.72)**
	1.8°C	1.31 (0.76, 2.25)	**5.09 (2.01, 12.88)**	**13.50 (4.28, 42.52)**
	14.8°C	1.65 (0.96, 2.82)	**5.69 (2.25, 14.41)**	**35.64 (11.64, 109.11)**
	24.0°C	1.69 (0.98, 2.94)	**5.45 (2.11, 14.07)**	**48.17 (15.46, 150.05)**
	26.4°C ^b^	1.71 (0.99, 2.97)	**5.40 (2.09, 13.93)**	**49.40 (15.86, 153.87)**
	30.9°C	**1.76 (1.01, 3.06)**	**5.47 (2.10, 14.20)**	**46.07 (14.39, 147.52)**
Female	-8.4°C	1.17 (0.92, 1.49)	**1.86 (1.25, 2.78)**	**2.83 (1.72, 4.65)**
	1.8°C	1.61 (0.90, 2.88)	**5.42 (2.01, 14.64)**	**18.29 (5.42, 61.73)**
	14.8°C	**2.01 (1.13, 3.57)**	**6.62 (2.45, 17.85)**	**39.24 (11.98, 128.53)**
	24.0°C	**2.33 (1.29, 4.22)**	**6.88 (2.50, 18.97)**	**57.00 (17.07, 190.36)**
	25.9°C^b^	**2.37 (1.31, 4.29)**	**6.85 (2.49, 18.87)**	**57.64 (17.27, 192.38)**
	30.9°C	**2.41 (1.32, 4.38)**	**6.62 (2.38, 18.36)**	**52.32 (15.20, 180.04)**
<1 year	-8.4°C	1.78 (1.04, 3.03)	1.56 (0.72, 3.37)	2.15 (0.80, 5.80)
	1.8°C	3.63 (0.99, 13.34)	3.19 (0.48, 21.26)	**11.99 (1.07, 134.91)**
	14.8°C	3.42 (0.94, 12.40)	5.20 (0.78, 34.61)	**54.78 (5.25, 572.00)**
	24.0°C	**4.53 (1.21, 16.97)**	**7.62 (1.10, 52.77)**	**116.72 (10.76, 1266.56)**
	25.4°C^b^	**4.59 (1.23, 17.17)**	**7.46 (1.08, 51.55)**	**119.86 (11.07, 1297.63)**
	30.9°C	**4.69 (1.24, 17.71)**	5.52 (0.79, 38.63)	**87.90 (7.77, 994.17)**
1–3 years	-8.4°C	1.16 (0.92, 1.47)	**2.00 (1.36, 2.93)**	**2.52 (1.58, 4.04)**
	1.8°C	1.53 (0.86, 2.70)	**5.95 (2.30, 15.44)**	**14.98 (4.73, 47.40)**
	14.8°C	1.73 (0.98, 3.04)	**6.43 (2.49, 16.60)**	**37.78 (12.29, 116.12)**
	24.0°C	**1.93 (1.08, 3.44)**	**7.17 (2.72, 18.88)**	**55.83 (17.85, 174.63)**
	27.5°C^b^	**1.98 (1.11, 3.53)**	**7.20 (2.74, 18.94)**	**58.08 (18.54, 181.99)**
	30.9°C	**1.99 (1.11, 3.58)**	**7.10 (2.68, 18.80)**	**56.53 (17.64, 181.15)**
3–6 years	-8.4°C	0.96 (0.75, 1.23)	**1.62 (1.05, 2.49)**	**2.19 (1.27, 3.76)**
	1.8°C	1.09 (0.59, 1.99)	**4.07 (1.38, 11.93)**	**11.33 (2.98, 43.05)**
	14.8°C	1.57 (0.86, 2.85)	**4.66 (1.58, 13.73)**	**26.10 (7.08, 96.15)**
	24.0°C	1.57 (0.85, 2.91)	**3.77 (1.25, 11.35)**	**31.70 (8.43, 119.17)**
	25.0°C^b^	1.58 (0.85, 2.92)	**3.73 (1.24, 11.22)**	**31.89 (8.49, 119.77)**
	30.9°C	1.65 (0.88, 3.08)	**3.81 (1.25, 11.57)**	**26.73 (6.84, 104.50)**
6–15 years	-8.4°C	1.46 (0.95, 2.25)	**3.50 (1.68, 7.27)**	**7.04 (2.77, 17.87)**
	1.8°C	2.33 (0.83, 6.57)	**21.86 (3.58, 133.60)**	**138.46 (14.33, 1337.37)**
	14.8°C	2.41 (0.87, 6.69)	**30.46 (4.99, 185.85)**	**286.26 (31.97, 2563.37)**
	24.0°C	**3.18 (1.10, 9.17)**	**43.58 (6.84, 277.71)**	**518.00 (55.28, 4853.93)**
	30.9°C	**3.13 (1.08, 9.11)**	**51.73 (8.03, 333.48)**	**646.66 (65.31, 6402.59)**
	34.5°C ^b^	**3.00 (1.01, 8.90)**	**61.00 (9.05, 411.40)**	**787.95 (70.38, 8821.03)**

^a^ -8.4°C,1.8°C, 14.8°C, 24.0°C and 30.9°C represent the 1st percentile, 25th percentile, 50th percentile, 75th percentile and 99th percentile of temperature in Beijing, respectively

^b^ The temperature that had the highest relative risk on total children, gender-specific and age-specific HFMD cases, respectively.

**Fig 6:**
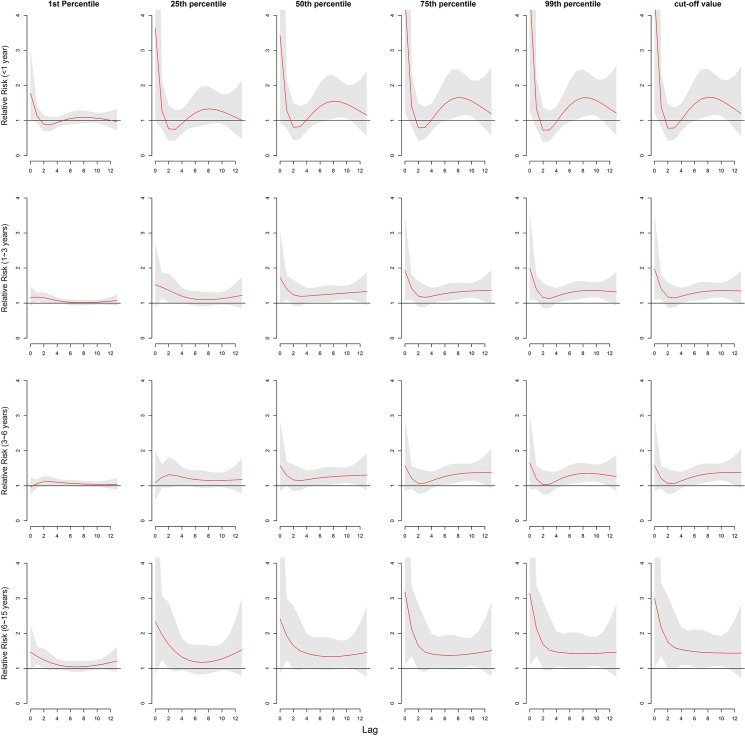
The relative risks of mean temperature (°C) for age-specific HFMD cases among different lags. (Note:1^st^ percentile:-8.4°C; 25^th^ percentile:1.8°C; 50^th^ percentile:14.8°C;75^th^ percentile:24.0°C; 99^th^ percentile:30.9°C; cut-off value for children aged less than 1 year:25.4°C; cut-off value for children aged 1~3 years: 27.5°C; cut-off value for children aged 3~6 years: 25.0°C; cut-off value for children aged 6~15 years: 34.5°C.)

Gender-specific RRs were noticed where temperature had the larger risk estimates of HFMD incidence in females than in males (Table 3). The associations of temperatures with HFMD for males lasted longer than for females (Fig 5). For age-specific RRs, it is the largest for children aged 6–15 years and smallest for those aged 3–6 years (Fig 6, Table 3). The associations of low temperatures and high temperatures with HFMD both occurred immediately for children aged <1 year (Fig 6). The associations of high temperatures with HFMD appeared immediately and the low temperatures did at lag 1 day for children aged 1–3 years and those aged 6–15years (Fig 6). For children aged 3–6 years, the risks for temperature occurred later than the other aged group, with the risks due to high temperatures occurring earlier that low temperatures (Fig 6).

Sensitive analyses indicated that when the maximum lag was changed from 13 to 20 days or the dfs for weather variables, similar exposure-response curve and cut-off temperature were obtained.

## Discussion

In this study, we examined the association of temperature with gender- and age-specific HFMD among children in Beijing, China, during 2010–2012. This is the first study that assessed the associations of delayed and cumulative exposure to temperature with gender- and age-specific HFMD by using a case-crossover design combined with DLNM. There were non-linear relationships between temperature and the incidence of children’s HFMD, with the largest RRs at 25.0~27.5°C. In general, the risks due to high temperatures were higher, occurred earlier and lasted longer than the low temperatures for HFMD cases. Girls and those aged 6–15 years were more vulnerable to the temperature. The results may provide a basis for preventing and controlling the incidence of HFMD based on temperature. The information may have implications for the early warning and response system on the disease.

A seasonal pattern was reported in our study with the peak in late spring (May) and early summer (June-July), followed by a second small peak only occurred in late autumn (November) of 2011. These patterns were similar to those found in several Asian countries (Malaysia, Singapore, Taiwan, Hongkong) as well as the UK [3, 4, 8, 22, 23]. However, in Finland most HFMD cases were reported in autumn [9]. Therefore, the seasonal pattern was inconsistent in different areas and even during different study periods in the same area. One explanation of the discrepancy was that the meteorological factors might influence the seasonal variation of HFMD cases but the mechanism has not become clear [10]. In addition, a laboratory-based study found Coxsackie B viruses peaked in June while Echoviruses showed two peak periods: June-July and September-October [24]. This indicated that the seasonal patterns of HFMD may depend on the type of virus. In short, the inconsistent seasonal patterns suggested that early warnings systems should be made based on the epidemics pattern of the target area and the type of virus.

We found a greater association of ambient temperature with HFMD for females than for males. To date, few studies have explored the gender-specific impact of temperature on HFMD incidence. Chen et al (2013)[15] reported that there was no significant difference between males and females in Guangzhou. Previous research examining the high temperature on mortality also found the mixed gender-specific temperature effects [25–27]. The inconsistency of vulnerability to temperature in male and female children might be due to the different socioeconomic characteristics of families and adaptive capacity of children at different locations. The age-specific results showed that children aged 6–15 years and <1 year were most vulnerable to temperature. This may be partly because the group aged 6–15 years play outdoors more often than younger children, even during higher temperature days. Ooi et al (2002) [28] reported that the level of maternal antibodies to EV71 in the infants waned one month after birth, so the lack of immunity increased the susceptivity of children aged <1 year to the temperature.

We observed a non-linear relationship between temperature and HFMD incidence. An interesting finding is that the exposure-response curve is an approximately inverted V-shape, which means that the relative risks decreased when temperature was higher than the cut-off temperature. In our study, the gender and age groups had different peak RRs with at the temperature range of 25.0~27.5°C. A study in Taiwan also produced an inverted V-shaped relationship between temperature and incidence of EV71 infections [14]. A study in Guangzhou found that RRs increased when temperature was below 25°C but became relative flat when temperature was above 25°C in the exposure-response curve [13]. Most previous studies reported a linear relationship between temperature and HFMD [10, 12, 15]. There are several reasons for this inconsistency. Firstly, we further explored the association of different temperature with HFMD at lag days and found that the cut-off temperature had longer lag effects, resulting in larger overall effects than other temperatures. However, most studies examined the association of temperature on HFMD at a weekly scale, which may miss detailed shorter-term information. Besides, other models used by previous studies, such as multiple linear regression model and generalized additive model with single-week or moving average lags, might not adequately capture the overall RRs of temperature on HFMD. Furthermore, it was possible that temperature could influence the dynamics of the infection transmission by affecting the survival and transmission of the HFMD virus, as well as the behaviour and activity of the participants [15]. Higher temperature may reduce the outdoor activities of children, thereby decreasing the contacts with other children [29]. This partly explained why the peak point on the exposure-response curve existed.

For the lag effects, in general, the risks due to low temperatures occurred later and were shorter while the risks due to high temperatures occurred immediately and lasted longer. The finding may be related to the fact that temperatures can have influence on the development and longevity of the virus [14, 30]. For instance, the virus could reproduce more rapidly and survive for longer at high temperature than at low temperature, so the occurrence of HFMD cases was immediate and lasted longer days at high temperature. So far, few data are available on this association of delayed exposure to daily temperature with HFMD and it needs further investigation.

A few limitations of this study should also be acknowledged. First, we used the ambient temperature data from a fixed site rather than individual exposure, so there may be some inevitable measurement error. Second, this study focused on only one city and the result from this study may apply to the cities with the similar temperature zone but not necessarily to the cities with different temperature zone.

In conclusion, our study reveals a non-linear association between daily ambient temperature and children’s HFMD. A temperature range of 25.0–27.5°C was expected to generate the highest relative risks for HFMD cases. The high-temperature risk was greater than the low-temperature. Female children and those aged 6–15 years were more vulnerable to the temperature. Further studies are required to confirm these findings in other populations.
